# Advances in Cereal Crops Breeding

**DOI:** 10.3390/plants10081705

**Published:** 2021-08-19

**Authors:** Igor G. Loskutov

**Affiliations:** Federal Research Center the N.I. Vavilov All-Russian Institute of Plant Genetic Resources (VIR), St. Petersburg 190000, Russia; i.loskutov@vir.nw.ru

Cereals are the main food and feed crops on our planet, with wheat, rice, and maize occupying three-quarters of the total acreage. The vast majority of plant breeders and plant geneticists around the world are engaged in cereal breeding. The genetic resources for crop genepools, including breeding and research materials, landraces, and wild crop relatives, which collectively are the pillars of modern plant breeding, are maintained ex situ in gene banks. The main challenges or bottlenecks in the advanced breeding techniques currently used in cereals are connected with concerns related to climate change, with breeding programs aiming to increase yield and tolerance to biotic and abiotic stresses (e.g., yield potential and resistance to main diseases and pets, as well as increased drought, heat tolerance, and nutrient efficiency). In the last few years, a trend has occurred in cereal crop breeding aimed at combining high agronomic and biochemical parameters in a single cultivar. Currently, traditional genetic and innovative molecular genetic methods are widely used in the breeding of grain crops. The success of biotechnology approaches has expanded the breeding possibilities and allowed interspecies and intergenus hybrids to be obtained. The development of molecular biology and genomics has completely overcome the barriers limiting the breeding of living organisms, while methods for genome editing of agricultural crops are still being improved to achieve higher levels of accuracy. Studies aimed at finding genes and quantitative traits loci (QTLs) that affect the main breeding traits and at identifying the desired allelic variants are currently relevant. In the field of genetic sequencing, genotyping by sequencing, also called GBS, is a method used to discover single-nucleotide polymorphisms (SNP) in order to perform genotyping studies, such as genome-wide association studies (GWAS).

The acquisition of large-scale phenotypic data has become one of the major bottlenecks hindering crop breeding and functional genomics studies. Nevertheless, recent technological advances have provided potential solutions to relieve such bottlenecks and to explore advanced methods for large-scale phenotyping, data acquisition, and data processing in the coming years. The phenomics data generated are already beginning to be used to identify genes and QTL through QTL mapping, association mapping, and genome-wide association studies (GWAS), in order to achieve crop improvements through genomics-assisted breeding (GAB). There is no doubt that accurate high-throughput phenotyping platforms will accelerate improvements in plant genetics.

This Special Issue on ‘Advances in Cereal Crops Breeding’ comprises 9 papers covering a wide array of aspects, ranging from the expression-level investigation of genes in terms of salinity stress adaptations and their relationships with proteomics in rice, the use of genetic analysis to assess the general combining ability (GCA) and specific combining ability (SCA) in promising hybrids of maize, the use of DNA markers based on PCR in rice, the identification of quantitative trait loci (QTLs) in wheat and simple sequence repeats (SSR) in rice, the use of single-nucleotide polymorphisms (SNP) in a genome-wide association study (GWAS) in cereals, and Nanopore direct RNA sequencing of related with LTR RNA retrotransposon in triticale prior to genomic selection of heterotic maize hybrids.

In order to better understand the mechanisms involved in salinity stress adaptations in rice, two contrasting rice cultivars were compared in a recent study—Luna Suvarna, a salt-tolerant cultivar, and IR64, a salt-sensitive cultivar. The expression-level investigation of auxin signaling pathway genes revealed increases in the transcript levels of several auxin homeostasis genes in Luna Suvarna compared with IR64 under salinity stress. Furthermore, protein profiling showed 18 proteins that were differentially regulated between the roots of two cultivars, some of which were salinity-stress-responsive proteins found exclusively in the proteome of Luna Suvarna roots, revealing the critical role of these proteins in imparting salinity stress tolerance. The results show that Luna Suvarna involves a combination of morphological and molecular traits of the root system that could prime the plant to better tolerate salinity stress [1].

The tolerance of rice to salinity stress involves diverse and complementary mechanisms, such as the regulation of genome expression, activation of specific ion transport systems to manage excess sodium at the cell or plant level, and anatomical changes that mitigate sodium penetration into the inner tissues of the plant. The identification of salinity tolerance QTLs associated with different mechanisms involved in salinity tolerance requires the greatest possible genetic diversity to be explored. In the investigation of genotyped rice landraces, SNP markers were used, with the aim of identifying new QTLs involved in salinity stress tolerance via a genome-wide association study (GWAS). Twenty-one identified QTLs colocalized with known QTLs. Several genes within these QTLs have functions related to salinity stress tolerance and are mainly involved in gene regulation, signal transduction, and hormone signaling. This study provides promising QTLs for breeding programs to enhance salinity tolerance and identifies candidate genes that should be further functionally studied to better understand salinity tolerance mechanisms in rice [2].

In addition to water flooding and salinity, rice growers in some parts of the world are also facing drought; thus, developing new rice genotypes tolerant to water scarcity is one of the best strategies to maximize yield potential and achieve water savings. In a recent study, rice genotypes were characterized for grain and agronomic parameters under normal and drought stress conditions and genetic differentiation was determined via specific DNA markers related to drought tolerance using simple sequence repeats (SSR) and cultivar grouping, establishing their genetic relationships with different traits. All genotypes were grouped into two major clusters with 66% similarity based on Jaccard’s similarity index. As a result of the study, genotypes were identified that could be included as appropriate materials for developing a drought-tolerant breeding program. Genetic diversity is needed to grow new rice cultivars that combine drought tolerance with high grain yields, which is essential to maintaining food security [3].

Recent studies on the tolerance to biotic and abiotic stressors in rice hybrids with donor lines of the genes of interest showed the effectiveness of such hybrids. As a result of the studies carried out using molecular marking based on PCR in combination with traditional breeding, early-maturing rice lines with genes resistant to salinity (SalTol) and flooding (Sub1A) were obtained, which are suitable for cultivation in southern Russia. The development of resistant rice varieties and their introduction into production will allow us to avoid the epiphytotic development of the disease, preserving the biological productivity of rice and resulting in environmentally friendly agricultural products [4].

The combining ability and genetic diversity of plants are important prerequisites for the development of outstanding hybrids that are tolerant to high plant density. A recent study was carried out to assess general combining ability (GCA) and specific combining ability (SCA), identify promising hybrids, estimate genetic diversity among the inbred lines, and correlate genetic distance (GD) to hybrid performance and SCA across different plant densities. As a result, no significant correlation was found between GD and either hybrid performance or SCA for grain yield and other traits, proving to be of no predictive value. Nevertheless, SCA could be used to predict hybrid performance across all plant densities. Overall, this study presents useful information regarding the inheritance of maize grain yield and other important traits under high plant density [5].

In addition to studying the productivity of plants and genes associated with general adaptability, the genetic improvement of root systems is of interest as an efficient approach to improve the yield potential and nitrogen use efficiency (NUE) of crops. QMrl-7B is a major stable quantitative trait locus (QTL) controlling the maximum root length in wheat. Two types of near isogenic lines (A-NILs with superior and B-NILs with inferior alleles) were used to specify the effects of QMrl-7B on root, grain output, and nitrogen-related traits under both low-nitrogen (LN) and high-nitrogen (HN) environments. The QMrl-7B A-NILs manifested larger root systems compared to the B-NILs, which is favorable to N uptake and accumulation, and eventually enhanced grain production. This study provides valuable information for the genetic improvement of root traits and breeding of elite wheat varieties with high yield potential [6].

Traditional plant breeding approaches supplemented with SNP markers used for genome-wide associative studies (GWASs) and genetic editing, as well as high-throughput chemotyping techniques, are exploited to speed up the breeding of desired genotypes. To enrich cereal grains with functional components, the new breeding programs need a source of genes in order to improve the contents of the beneficial components. The sources of these valuable genes are plant genetic resources deposited in genebanks, including landraces, rare crop species, and even wild relatives of cultivated plants. Correlations between the contents of certain bioactive compounds and the resistance to diseases or tolerance to certain abiotic stressors suggest that breeding programs aimed at increasing the levels of health-benefiting components in cereal grain might at the same time allow the development of cultivars adapted to unfavorable environmental conditions [7].

Using Nanopore long-term forward RNA sequencing, functionally important but unexplored RNA molecules have been identified, including long non-coding RNAs (lncRNAs), as they are often associated with repeat-rich regions of genomes and transposon-derived transcripts expressed during early stages of seed development in triticale. Detailed analysis of the protein-coding potential of the RTE-RNAs showed that 75% of them carry open reading frames (ORFs) for a diverse set of GAG proteins, the main components of virus-like particles of LTR retrotransposons. This demonstrated experimentally that certain RTE-RNAs originate from autonomous LTR retrotransposons, with ongoing transposition activity during early stages of triticale seed development. Overall, these results provide a framework for further exploration of the newly discovered lncRNAs and RTE-RNAs in functional and genome-wide association studies in triticale and wheat. The results also demonstrate that Nanopore direct RNA sequencing is an indispensable tool for the elucidation of lncRNA and retrotransposon transcripts [8].

Genomic selection (GS) shows great promise in terms of strongly increasing rates of genetic improvement in plant breeding programs. It allows for comparative larger gains from selection by estimating all marker effects simultaneously, while the subsequent selection of genetically superior individuals is based on their genomic estimated breeding value (GEBV) instead of using a few significant markers, as is the case in classical marker-assisted selection (MAS). GS is ideal for complex traits with lower heritability and complex genetic architectures.

Genomic selection (GS) can accelerate variety improvement when the training set (TS) size and its relationship with the breeding set (BS) are optimized for the prediction accuracies (PAs) of genomic prediction (GP) models. Sixteen GP algorithms were run on phenotypic best linear unbiased predictors (BLUPs) and best linear unbiased estimators (BLUEs) of resistance to both fall armyworm (FAW) and maize weevil (MW) in a tropical maize panel. Random-based training sets (RBTS) and pedigree-based training sets (PBTSs) were designed to study biotic resistance. For PBTS, the FAW resistance PAs were generally higher than those for RBTS, except for one dataset. GP models generally showed similar PAs across individual traits, whilst the TS designation was determinant, since a positive correlation between TS size and PAs was observed for RBTS, while for the PBTS, this correlation was negative. The resulting population could be of interest in future breeding activities targeted at improving insect resistance in maize and could be potentially useful for GS of complex traits with low to moderate heritability. This study has pioneered the use of GS for maize resistance to insect pests [9].

Advances in cereals breeding to develop new improved cultivars are some of the most important factors in agricultural production, playing an essential role in ensuring sustainable agriculture. Along with classical breeding goals, innovative, modern plant breeding methodologies are applied here to create new cultivars of crops for current and future agriculture applications. This endeavor includes the development of cultivars for stress cultivation conditions to achieve sustainable agricultural production, increased food quality, and increased security, and to supply raw materials for innovative industrial products and to meet the needs of mankind.
